# Proteome-wide analysis of protein stability in *Escherichia coli* under acid stress

**DOI:** 10.1093/jimb/kuag016

**Published:** 2026-07-01

**Authors:** Onyeka Onyenemezu, Jeffrey N Law, Jetendra K Roy, Chao Wu, Joel Nott, Robert L Jernigan, Laura R Jarboe

**Affiliations:** Department of Chemical and Biological Engineering, Iowa State University, Ames, IA 50011, United States; Biosciences Center, National Laboratory of the Rockies, Golden, CO 80401, United States; Department of Chemical and Biological Engineering, Iowa State University, Ames, IA 50011, United States; Biosciences Center, National Laboratory of the Rockies, Golden, CO 80401, United States; Protein Facility, Iowa State University, Ames, IA 50011, United States; Department of Biochemistry, Biophysics and Molecular Biology, Iowa State University, Ames, IA 50011, United States; Department of Chemical and Biological Engineering, Iowa State University, Ames, IA 50011, United States

**Keywords:** label-free, protein solubility, stability, enzyme assay

## Abstract

Knowledge of protein acid sensitivity remains sparse and is largely derived from low-throughput, enzyme-specific assays. We used a scalable framework to map acid stability across the *Escherichia coli* proteome to assess the acid stability of 1,675 unique proteins, estimating pH_50_ values for over 90% of them. The parameter pH_50_ was defined as the pH value at which only 50% of the initial protein remains in solution following acid treatment. Proteome-wide pH_50_ values ranged from 2.28 to 6.33 (median 5.11). Approximately 9% of detected proteins remained stable across all tested pH conditions. Our results align with published data and the assay of citrate synthase (GltA) performed here. Protein acid stability differed significantly by subcellular localization: periplasmic proteins were relatively more abundant in the acid-stable group, cytoplasmic proteins were abundant at pH_50_ values 4.5–5.5, and inner membrane proteins at higher pH_50_ between 5.5 and 6.0. Outer membrane proteins were too few to draw strong conclusions regarding enrichment within specific pH_50_ groups. Notably, the periplasmic binding protein of the molybdate ABC transporter (ModA), was enriched after incubation at low pH. Estimated pH_50_ values showed no correlation with protein isoelectric point and molecular weight. Together, this work provides the first proteome-wide map of protein acid stability and establishes a general framework for studying different chemical stressors.

**One sentence summary** This paper presents a quantitative map of acid stability of *E. coli* proteins and established a procedure for the study of protein acid sensitivity that is adaptable to other stressors and other organisms.

## Graphical Abstract

**Graphical Abstract:**
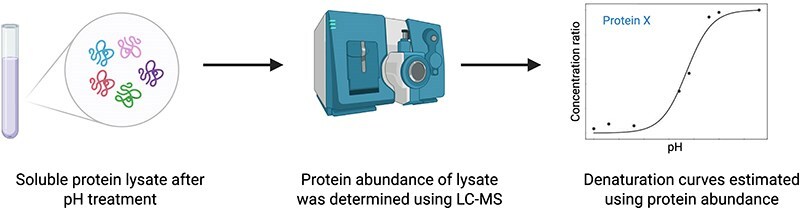

## Introduction

Proteins are the principal functional molecules of living systems, directly executing nearly all biological processes. However, these intricately folded amino acid polymers are sensitive to environmental conditions such as pH, temperature, and the presence of solvents. One key property governing a protein’s function is stability, which is important to maintain structure and functionality in the presence of various stressors, such as acid that is being considered here. Lower stability can lead to unfolding, aggregation, and increased susceptibility to proteolysis (Fágáin, 1995; Jaenicke, 1991).

Improving protein stability can help in the optimization of cell-free biosynthesis platforms (Batista et al., 2021), storage of protein products (Parnian et al., 2026; Rahban et al., 2023), and inform enzyme engineering strategies (Jiang et al., 2023). For example, broad and deep knowledge of protein thermostability helps increase the robustness of bioprocesses, improves the efficiency of therapeutic and industrial enzymes (Nezhad et al., 2022; H. Wu et al., 2023), and enables development of comprehensive sequence- and structure-based models (Pinney et al., 2021; Yang et al., 2022). However, quantitative knowledge of other aspects of protein stability, such as at low pH, is not readily available.

Traditional stability assays rely on enzyme-specific activity measurements performed under heterogeneous experimental conditions, often requiring bespoke substrates and protocols. While these assays provide valuable insight for individual enzymes, low throughput and lack of standardization limit useful comparisons across proteins and studies. As a result, organism-wide conclusions about protein stability cannot be readily drawn from existing data. This limitation is underscored by the scarcity of published stability measurements: among the approximately 4,200 proteins encoded by the *Escherichia coli* genome, pH stability assay data are available for only a few dozen proteins (Table S1), highlighting the sparse data problem for any broad proteome-level understanding of acid stability.

Proteomics-based approaches offer a powerful alternative by enabling the simultaneous analysis of thousands of proteins under uniform conditions to investigate the loss of solubility associated with destabilization. Previously, thermal proteome profiling and related methods have provided valuable insights into protein thermostability and proteome-wide melting behavior (Jarzab et al., 2020; Le Sueur et al., 2022; Leuenberger et al., 2017; Mateus et al., 2018). However, to the best of our knowledge, all proteome-wide stability analyses to date have focused on temperature, leaving other physiologically relevant stressors comparatively unexplored.

Acidity represents a fundamentally distinct and biologically important challenge to protein stability. Unlike temperature, which affects both intracellular and extracellular environments in approximately uniform ways, bacteria actively maintain their cytoplasmic pH within a narrow range, despite fluctuations in extracellular pH (Krulwich et al., 2011; Lund et al., 2014; O’Sullivan & Condon, 1997; Padan et al., 1981; Padan & Schuldiner, 1986; Slonczewski et al., 2009). For example, multiple acidophilic microbes have been observed to maintain an intracellular pH of 4.5 or higher when grown in media at pH ∼ 1 (Slonczewski et al., 2009). In contrast, even when the external environment is maintained at a neutral pH, the presence of organic acids can result in intracellular acidification (Royce et al., 2014). Thus, there is a decoupling between the readily-observed external pH and the less accessible cytoplasmic pH. This distinction complicates the relationship between environmental acidity and the stability of proteins expressed by the associated organism(s), making acid stress a nontrivial and underexplored dimension of proteome behavior. Moreover, pH-dependent stability is directly relevant to protein storage, downstream processing, and importantly large-scale fermentation, where acidification often negatively impacts enzyme function, cellular viability, and bioprocess productivity.

**Figure 1:**
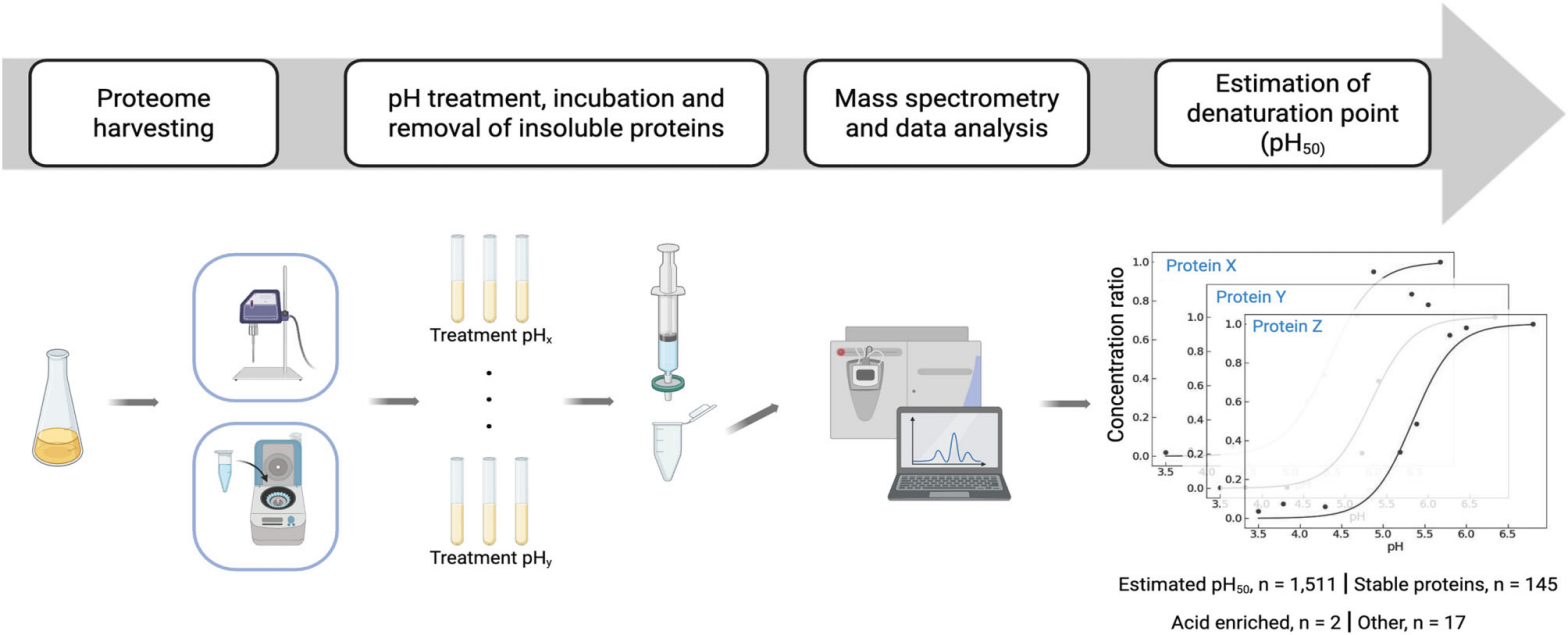
Experimental workflow for assessing proteome acid stability.​ *Escherichia coli* cells were grown in rich medium initially at neutral pH. Harvested cells were sonicated in lysis buffer with protease inhibitors and the resulting lysates were incubated across a range of acidic pH values for 48 hr at 4 °C. Insoluble proteins were then removed and the remaining soluble proteins were analyzed by liquid chromatography–mass spectrometry (LC-MS). The stability of each protein was quantified by estimating the pH at which the concentration of the protein was 50% of initial value (pH₅₀). The solid line on the graph represents the best fit line for protein abundance to the sigmoidal equation.

Here, we present a proteomics-based framework for the systematic analysis of protein acid stability, using *E. coli* as a model organism (Figure 1). We establish and validate standardized protocols for sample preparation and data analysis to enable comparative, proteome-wide assessment of protein stability under acidic conditions. By extending stability profiling beyond temperature to acidity, this study expands the current understanding of protein stability at the systems level and provides a foundation for the type of quantitative analysis that can contribute to identification of unstable protein targets for engineering.

## Methods

The protocol used in this study has been adapted from previous thermostability work (Jarzab et al., 2020; Leuenberger et al., 2017; Mateus et al., 2018), with the modification that protein lysate are incubated at different pH values instead of different temperatures. Proteomic analysis was performed at the Iowa State University Protein Facility.

### Organism growth and preparation of cell lysate

All growth experiments used *E. coli* K12 MG1655. Cells were grown in filter-sterilized BD Difco^TM^ Luria–Bertani broth (LB) medium (initial pH 7.0) in 250 ml baffled flasks filled to 50 ml at 37 °C with rotary shaking at 250 rpm to an OD_600_ of ∼0.8. Cells were harvested by centrifugation at 4000 x *g* for 10 min at 4 °C and washed with ice-cold 1X phosphate buffered saline (PBS) buffer (pH 7.0), pelleted again, and then resuspended in the lysis buffer. The lysis buffer contained one tablet of Complete Protease Inhibitor (Roche #65 726 900) in 50 ml 1X PBS (pH 7.0). The cells were then sonicated at 35% amplitude at 4 °C, with cycles of pulse on for 5 s, pulse off for 30 s, for approximately 18 min. The resulting lysates were then centrifuged at 4000 x *g* for 20 min at 4 °C to remove cellular debris. The supernatants were transferred to a fresh tube, and the protein concentration was determined using the Modified Lowry Protein Assay Kit (Thermo Scientific). Protein concentration was then adjusted to approximately 4 mg/ml using the lysis buffer, generating a stock proteome solution.

### Acid treatment of cell lysate

Ice-cold lysis buffer was prepared as above, but with varying pH values, adjusted using HCl. The stock proteome solution was added to a target concentration of 1 mg/ml. Samples were held for 48 hr at 4 °C after which the pH of each mixture was measured; these measured values are the pH values reported throughout this study. Samples were then filtered through a 0.45-µm low protein-binding syringe filter (Millipore, SLHVR13SL), and the concentration of remaining soluble proteins was estimated using the Lowry assay.

### SDS-PAGE

Each of the soluble fractions were denatured with Laemmli sample buffer (Bio-Rad #1 610 737) containing 5% v/v β-mercaptoethanol and heated for 8 min at 98 °C and separated on sodium dodecyl sulfate (SDS)‐polyacrylamide precast gels (Bio-Rad #4 561 083) by electrophoresis at 120 V. The gels were stained with Bio-Rad Coomassie Brilliant Blue and de-stained according to standard protocols.

### Protein digestion

Soluble fractions were reduced at 37 °C for 30 min with 5 mM dithiothreitol and alkylated with 15 mM iodoacetamide for 30 min at room temperature in the dark. The solution was then digested overnight at 37 °C with trypsin/Lys-C at a ratio of 1 μg trypsin/Lys-C:25 μg sample. Formic acid was added to a 1% v/v concentration to stop digestion, and the digestion was dried in a SpeedVac. Samples were then desalted using C18 columns (Nest Group BioPureSPN Mini, HUM S18V) before drying again in a SpeedVac. Pierce Peptide Retention Time Calibration Mixture (PRTC, #88 320) was spiked into the sample at 25 fmol/μl to serve as an internal control.

### LC-MS/MS analysis

Peptides were separated using a Thermo Scientific DNV PepMap Neo (75 μm × 50 cm, double nano viper, DNV75500PN) with a stainless-steel emitter. A linear gradient from Buffer A (0.1% v/v formic acid in water) and Buffer B (80% v/v acetonitrile, 20% water, and 0.1% v/v formic acid) was applied over 120 min at a flow rate of 0.2 μl/min. The peptide mixture was run on a Thermo Scientific EASY-nLC 1200 coupled to a Thermo Scientific Nanospray FlexIon source and MS/MS was run on a Thermo Scientific Q Exactive Hybrid Quadrupole-Orbitrap Mass Spectrometer with a Higher-Energy Collisional Dissociation (HCD) fragmentation cell.

The analysis, protein, and peptide quantification were performed on Proteome Discoverer v.3.0. The data were searched using Mascot and Sequest HT against the Uniprot *E. coli* and PRTC databases. Proteome Discoverer’s Minora Feature Detection node was used for the label-free quantification. The mass tolerance for chromatographic alignment was set to ±10 ppm, and results were adjusted to a 1% protein false discovery rate. Carbamylation (Cys) was set as a fixed modification, with dynamic modifications of oxidation (Met) and deamidation (Asn, Gln).

### Estimation of the denaturation point (pH_50_) and confidence interval

**Figure 2:**
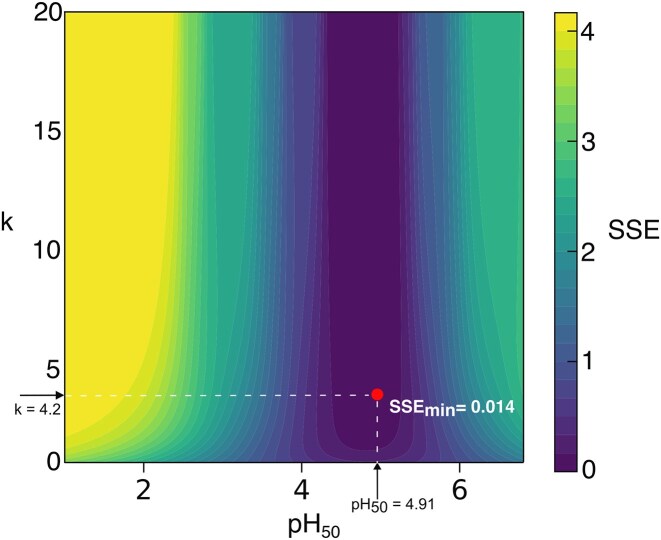
A grid map was used to estimate pH₅₀. The grid map illustrates the SSE for all combinations of *k* (the slope of the sigmoid curve, mg/ml per pH) and pH_50_ evaluated for GadA protein. The red point corresponds to the lowest SSE and therefore the best *k* and pH_50_ values that fit the data to the model.

Normalized soluble protein concentration was assumed to trend with treatment pH and protein denaturation point (pH_50_) as described in Equation 1. Data acquired across multiple pH values was fit to Equation 1 by performing a two-dimensional grid search across a range of values of the slope parameter (*k*) and pH_50_ values. Values of *k* and pH_50_ were sampled between 1 and 20 and 1.0 and 6.8, respectively. For each pair of *k* and pH_50_ values, the model was evaluated using the normalized concentration across pH measurements, and the sum of squared errors (SSE) between observed and predicted values was computed. The pair that produced the lowest SSE (SSE_min_) across the entire grid was selected as the best-fit estimate (Figure 2).


(1) \begin{eqnarray*}
f\left( {pH} \right) = \frac{1}{{1 + {{e}^{ - k\ \left( {pH - \ p{{H}_{50\ }}} \right)}}}}.
\end{eqnarray*}

The 95% confidence interval for each estimated pH_50_ value was defined as the range of pH values for which the SSE remained below a threshold SSE value (SSE_thresh_). SSE_thresh_ was computed using the chi-square distribution for the sample variance (S^2^) with significance level a of 0.05 (Equations 2 and 3) (Devore, 2012), where *n* is the number of observations (maximum value of 10) and we have two fitted parameters:


(2) \begin{eqnarray*}
SS{{E}_{\mathit{thresh}}} = \frac{{{{{\left( {n - 2} \right)}}^2}{{S}^2}}}{{\chi _{\left( {0.975,\,n - 2} \right)}^2}},
\end{eqnarray*}

(3) \begin{eqnarray*}
{{S}^2} = \frac{{SS{{E}_{min}}}}{{n - 2}}.
\end{eqnarray*}

All analyses were conducted in Python (version 3.13.1), with statistical computations implemented using the SciPy package (version 1.15.2).

### Accounting for experimental noise

We measured protein stability across 10 distinct pH conditions (2.5, 2.8, 3.5, 3.8, 4.3, 5.2, 5.4, 5.6, 6.0, and 6.8). Proteins that retained at least some stability across this pH range would be expected to have 10 associated normalized concentration values. However, proteins that have a complete loss of stability would be detected in fewer than 10 conditions. These expectations are tempered by experimental noise and the stochastic nature of finite sampling. For example, some proteins were detected in the near-neutral pH values of 6.8 and 6.0, not detected at moderate pH values, such as 5.8 and 5.6, but then were detected again at low pH values such as 3.5 and 2.8. Therefore, we implemented a sliding-window strategy of the sigmoidal model presented above to decrease the influence of this noise while preserving information.

For each protein, we required concentration data for at least three sequential pH values. As a first pass, we considered only the four least acidic pH treatments: 6.8, 6.0, 5.8, and 5.4, estimating a 95% confidence interval for the pH_50_ for each protein. The dataset was then expanded to include the next two values, 5.2 and 4.3 and a new 95% confidence interval was estimated. A similar analysis was performed with eight pH values and then 10. The optimal sliding window for each protein was selected based on a combined criterion of minimal SSE and maximal data coverage. There were 40 proteins that were not detected at pH 6.8 and yet were quantified for at least pH 6.0, 5.8, and 5.4. For these proteins, the concentrations were normalized relative to pH 6.0 and the sliding window strategy still utilized four, six, and eight data points, with the final window consisting of nine data points instead of 10.

### Comparison of estimated pH_50_ with stability assay data from literature

For assessment of the pH_50_ distribution, we collected pH stability data for proteins expressed by *E. coli, Enterobacter, Klebsiella, Serratia*, and *Salmonella*. WebPlotdigitizer version 4.8 (Marin et al., 2017) was used in the extraction of data from enzyme pH stability graphs.

### Cloning, expression, and purification of GltA (citrate synthase)

The *gltA* gene was amplified via polymerase chain reaction (PCR) using *E. coli* K12 MG1655 genomic DNA as the template and pDx_gltA_fw and pDx_gltA_rv primers (Table S2). The primers were designed to include homologous regions for seamless recombination into the pDx_mscarlet3_SYFP2 vector. The vector was linearized using primers pDx_vec_fw and pDx_vec_rv primers (Table S2). The PCR product and linearized vector were assembled using NEB Gibson Assembly mix following the manufacturer’s protocol. The recombinant plasmid was transformed into *E. coli* DH5α by heat shock at 42 °C for 2 min. Positive clones were confirmed by Sanger sequencing.

The amplified plasmid was then transformed into *E. coli* BL21 for protein expression. A single colony was inoculated into LB broth containing 50 µg/ml kanamycin and grown overnight at 37 °C with shaking at 250 rpm. The overnight culture was diluted 1:100 in fresh LB broth and grown to an OD_600_ of 0.6–0.8, at which point protein expression was induced with 10 mM L-rhamnose and grown at 20 °C and 150 rpm overnight.

Cells were harvested by centrifugation at 4000 × *g*, 10 min, 4 °C, and resuspended in lysis buffer (pH 7.0). The cell suspension was lysed by sonication at 4 °C with an amplitude of 75% for 15 min with cycle pulse on for 10 s and pulse off for 20 s. After centrifugation, the supernatant was purified using the HisTrap FF Crude column from Cytiva according to the manufacturer’s protocol. Eluted fractions were analyzed by SDS-PAGE.

### Activity and stability assay of GltA protein

The assay of GltA was carried out as previously described (Danson & Weitzman, 1973; Srere et al., 1963). The assay mixture contained 0.10 mM oxaloacetate, 0.15 mM acetyl-CoA, and 0.10 mM 5,5'-dithiobis- (2-nitrobenzoic acid) in either 100 mM sodium acetate or 100 mM potassium phosphate buffers. For the stability assay, roughly 1 mg of purified GltA protein was incubated in buffers at pH 4.0–7.1 at 4 °C for 24 hr then the remaining activity was measured at pH 7.1. Incubation at pH values of 5.5 or below were performed in sodium acetate buffer. The enzyme activity was measured as the formation of coenzyme A (CoASH) at a wavelength of 412 nm 25 °C.

### Statistical analysis of pH_50_ distribution by subcellular locations

Subcellular location data for *E. coli* K-12 proteins were sourced from UniProt (The UniProt Consortium et al., 2025) (accessed April 23, 2026). Proteins were binned according to estimated pH_50_ values and pairwise chi-square tests of independence were performed to compare the number of proteins across subcellular locations. To account for multiple hypothesis testing across bins and pairwise comparisons, *p*-values were adjusted using the Benjamini–Hochberg false discovery rate correction. Differences were considered statistically significant at an adjusted *p*-value of 0.05.

### Re-analysis of existing thermal stability data for *E. Coli* proteome

We reanalyzed the raw abundance data previously published by Jarzab et al., (2020) using our approach for the 95% confidence interval to predict a similar confidence interval for the melting point (*T_m_*). Data from their three biological replicates were merged and the median of the normalized fold ratios was used to fit the sigmoidal model (Equation 4):


(4) \begin{eqnarray*}
f\left( T \right) = \frac{1}{{1 + {{e}^{k\left( {T - Tm} \right)}}}}.
\end{eqnarray*}

Values of *k* were constrained between 1 and 20, as above, and values of *T_m_* were sampled between 37 and 67 °C.

### Correlation of pH_50_ with isoelectric point, molecular weight, and melting temperature

The theoretical isoelectric point (pI) and molecular weight (MW) for *E. coli* proteins were calculated based on UniProt amino acid sequences annotations. These properties were correlated with the estimated pH_50_ values determined in this study using the Pearson correlation. Protein melting temperature (*T_m_*) values were obtained from previously published proteome-wide thermostability datasets (Jarzab et al., 2020; Leuenberger et al., 2017; Mateus et al., 2018) and independently correlated with pH_50_ values.

## Results

To study the effect of pH on the *E. coli* MG1655 proteome, we adapted an approach developed to assess proteome thermostability (Jarzab et al., 2020; Leuenberger et al., 2017; Mateus et al., 2018). This approach exploits the insolubility of denatured proteins, resulting in their elimination from the sample prior to protein quantification and identification. Here, instead of treating the harvested proteome at various temperatures, we treated the harvested proteome at various pH values. Proteins that were not stable at the incubation pH became insoluble and were removed by filtration prior to mass spectrometric analysis (Figure 1).

### Increased acidity reduces protein solubility

**Figure 3:**
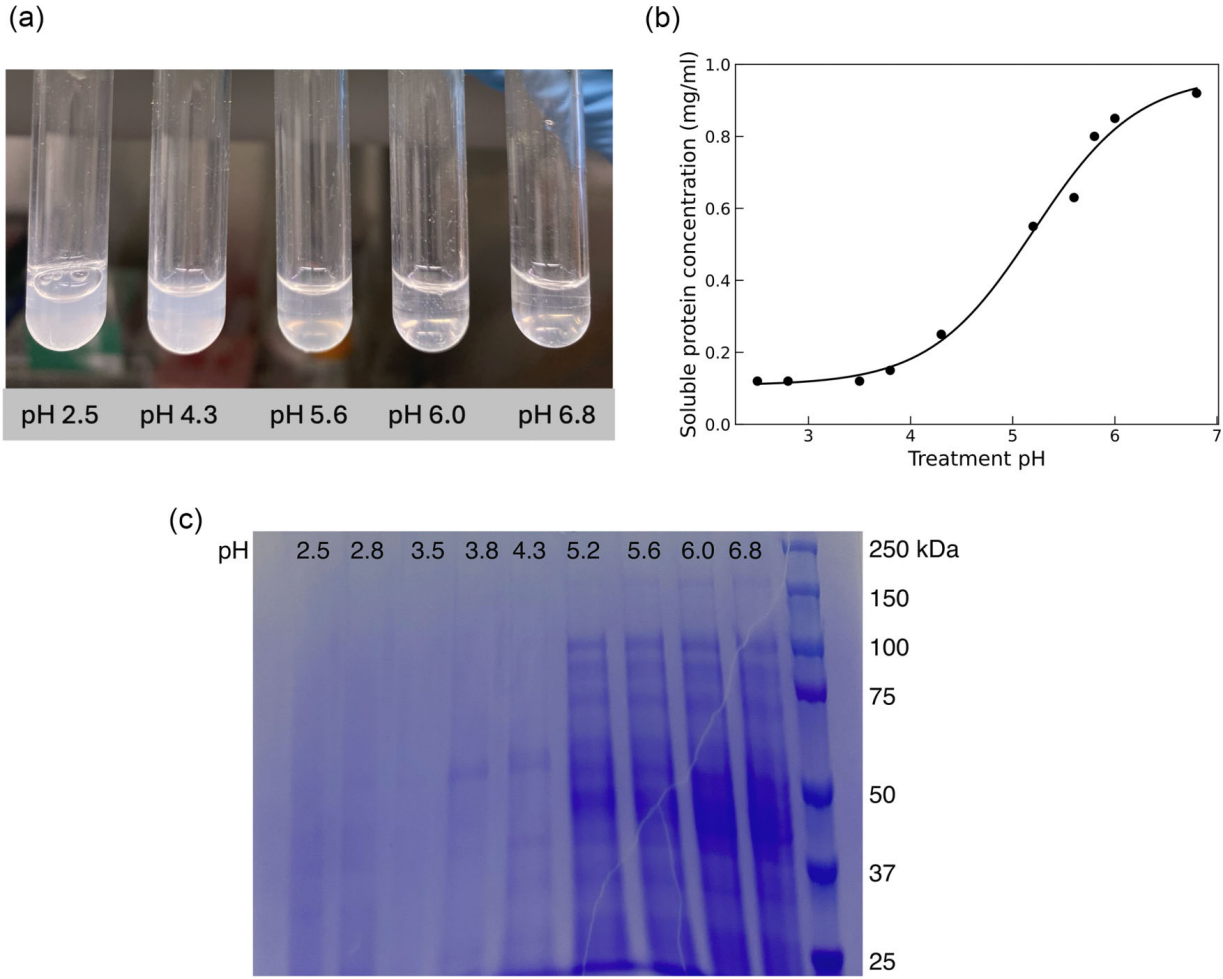
Acid treatment of *E.coli* lysates causes protein denaturation. ​Figure 3A: Visible precipitate forms at lower pH values illustrating that protein precipitation occurs under more acidic conditions. At neutral pH, the lysate remains clear, whereas acidification leads to formation of precipitates. ​Figure 3B: Soluble protein concentration decreases as a function of pH. After 48 hr of treatment, insoluble proteins were removed, and the soluble fraction quantified by a Modified Lowry assay.​ Figure 3C: Major protein bands progressively diminish below pH 5.2 due to widespread denaturation in strongly acidic conditions. Lanes are labeled with their respective pH values (top).​

Cells were grown to mid-exponential phase in rich media at pH 7.0 and lysed in the presence of a protease inhibitor cocktail. Lysates were then held at the indicated pH for 48 hr at 4 °C. This treatment resulted in the expected loss of protein solubility as a function of the treatment pH, evidenced by visible precipitation (Figure 3A). Similarly, the bulk concentration of soluble proteins decreased to less than 20% of the initial value at pH values lower than 4 (Figure 3B). Similar trends were apparent when treated lysates were visualized on a gel (Figure 3C). These results demonstrate the impact of pH on the bulk protein solubility.

The soluble protein fraction for each treatment pH was subjected to proteomic analysis (Table S3). The intention of our pH treatment was to enrich the soluble protein population based on the pH sensitivity of individual proteins. Of the proteins detected in solution at both pH 6.8 and 2.5, we observed a median concentration decrease of 84%. Also, of the 1,661 unique proteins identified in lysate treated at pH 6.8 475 (28.5%) decreased in concentration to such a degree that they were no longer detectable in the lysate treated at pH 2.5.

### Quantitative analysis

Having demonstrated that the “meltome” approach can be effectively modified for assessment of protein pH sensitivity, we normalized all the measurements of protein concentration. Specifically, the concentration of each protein in solution at each treatment pH was normalized relative to the baseline condition of pH 6.8. In a few cases (*n* = 40) where protein concentration was estimated at at least three pH values, but the protein was not detected at pH 6.8, concentrations were normalized relative to pH 6.0. Under ideal conditions, all normalized concentrations should be less than or equal to one. However, the inherent noise of this type of experimental procedure inevitably results in some values outside of this expected maximum. Specifically, out of 13,430 normalized protein concentrations, 20% are greater than one, with <1% exceeding five. The Jarzab meltome dataset (Jarzab et al., 2020) is similar, with 14% of the abundance ratios being greater than one. Thus, the noise in our dataset is consistent with other similar studies.

**Figure 4:**
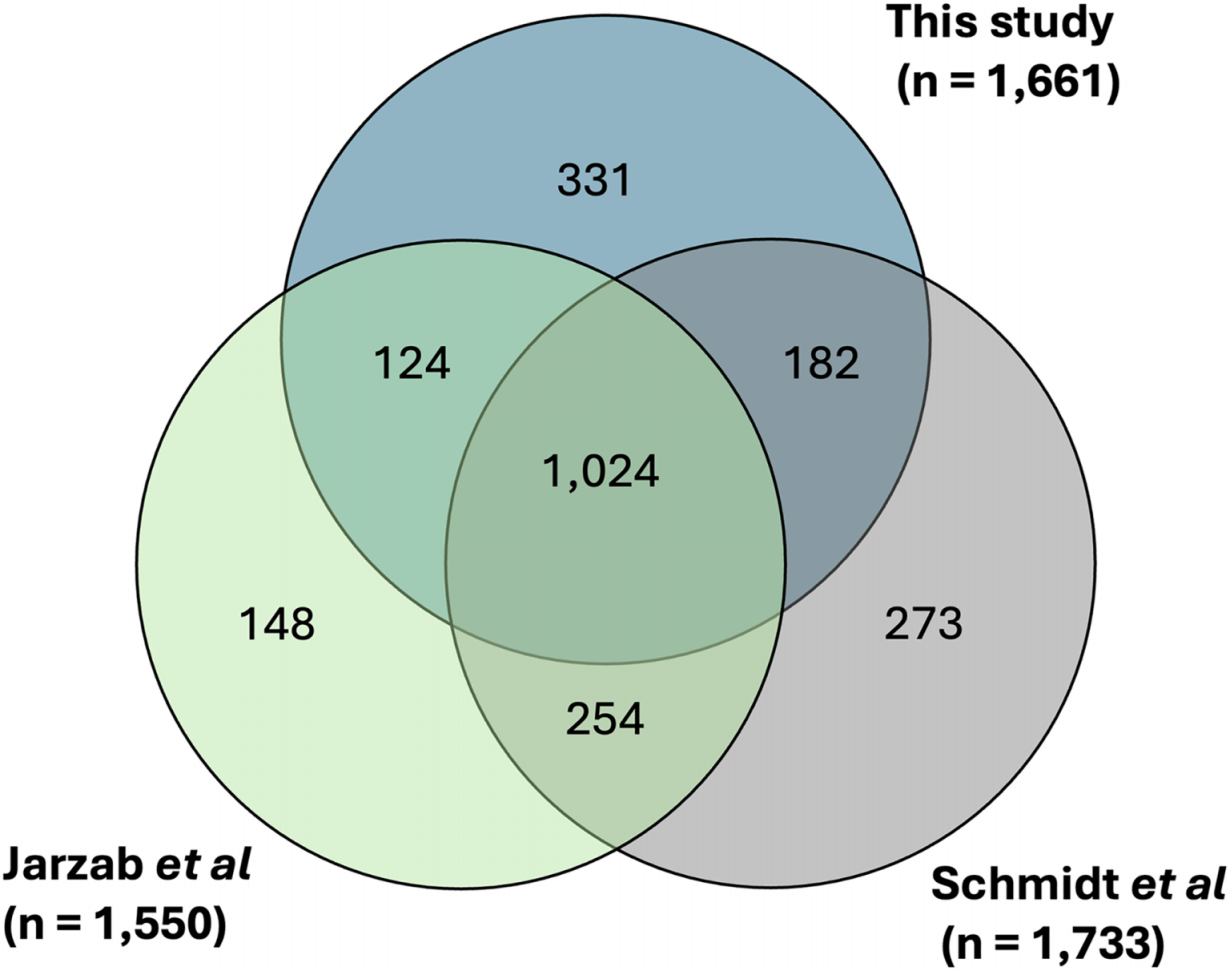
Comparison of proteins detected across studies. Venn diagram showing the overlap between the *E. coli* proteins analyzed in this work, those detected by Schmidt et al., and those subjected to thermal stability analysis by Jarzab et al. A core set of 1,024 proteins was observed in all three studies.

We detected 1,661 unique proteins at pH 6.8 (Figure 4). The growth conditions used here were similar to those used in Schmidt et al’s (2016) comprehensive proteomics study and Jarzab et al’s harvesting of proteins for assessment of thermal stability (Jarzab et al., 2020), which detected 1,733 and 1,550 respectively. Thus, the degree of coverage of the *E. coli* proteome is consistent with other studies. More than half of the proteins detected in each of these three studies were also detected in the other two. Note that the experimental procedure used here does not appear to be skewed against proteins known to be associated with the inner membrane, as our dataset and Schmidt’s consisted of 281 (16.9%) and 273 (15.8%) such proteins, respectively.

**Figure 5:**
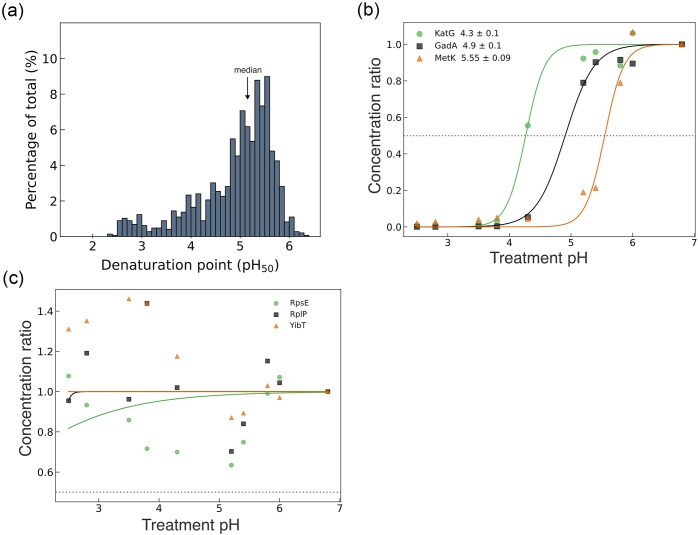
Scope of estimated pH_50_ values. Figure 5A: Frequency distribution shows that over 75% of proteins for which pH_50_ was estimated had pH_50_ above 4.5. Figure 5B: Denaturation curves of three exemplar proteins KatG, GadA, and MetK. Values are provided as the average estimate and the associated 95% confidence interval. Figure 5C: Profile for stable proteins showing the fitted sigmoidal curve at a concentration of one.

We then fit each of the normalized concentration profiles to a sigmoidal equation (Equation 1) and estimated the pH_50_ and the associated 95% confidence interval. We were able to estimate a pH_50_ value for 1,511 unique *E. coli* proteins (Table S4), ranging from 2.28 to 6.33 with a median of 5.11 (Figure 5A). We also calculated a 95% confidence interval for each estimated pH_50_ value. Across all proteins, the median size of this confidence interval is 0.33 pH units.

Data for three representative enzymes is shown in Figure 5B, with pH_50_ values above and below the median value. For example, MetK having a pH_50_ of 5.55 ± 0.09 means that it is more acid sensitive than KatG (pH_50_ 4.3 ± 0.1), with GadA having a pH_50_ that is intermediate to these two other proteins (pH_50_ 4.9 ± 0.1).

Surprisingly, some proteins showed evidence of sustained stability even in highly acidic treatment conditions. For example, RpsE, RplP, and YibT all had concentration ratios greater than 0.5 in all queried pH values (Figure 5C). Any protein that was detected in at least four treatments and had a concentration of at least 0.5 in all of these measurements was classified as ‘stable’ and removed from the data fitting pipeline. In total, 145 proteins were given this designation, representing 8.7% of the total proteins detected here. Note that these proteins are not represented in Figure 5A and do not contribute to the estimated median pH_50_ value. Recognition of the presumed structural stability of these enzymes at such low pH values is useful information for future studies.

Our workflow queried the acid stability of 1 675 unique *E. coli* proteins. For more than 90% of these, we calculated a pH_50_ value and the associated 95% confidence interval. We have classified roughly 9% of the proteins as stable. The remaining proteins are discussed below.

### Estimated proteome stability aligns with existing literature data on protein stability

As described above, many studies of protein pH tolerance assess activity as a function of pH and do not explicitly consider structural stability. Thus, there is only a small set of *E. coli* proteins previously characterized for pH stability. These assays typically involve incubation of purified protein at some pH value and temperature for some length of time, and then assessment of the remaining protein activity in a standard assay condition, typically near neutral pH. These stability assays lack a standard approach, with widely varying incubation times and temperatures. Therefore, our approach not only explicitly addresses protein stability, it also provides uniform assessment of proteome-wide stability.

We collected literature data describing the pH stability of 42 proteins from *E. coli* and other enteric bacteria (Table S1). These literature pH_50_ values had a median of 4.65 with 73% of the pH_50_ values between pH 4 and 6 in contrast to a median of 5.11 and 83%, respectively, for the results presented here (Figure S1). The distribution of the literature pH_50_ values does not significantly differ from the distribution of the *E. coli* proteome pH_50_ values reported here (unpaired *t*-test, *p* = 0.40).

We identified 18 *E. coli* enzymes with quantitative assessment of pH stability data available both in the literature (Aisaka et al., 1991; Baldwin & Davidson, 1983; Berman & Cohn, 1970; Burns & Demoss, 1962; Cozzani et al., 1980; Donovan & Kushner, 1983; Furukawa et al., 2014; Gushima et al., 1983; Hayashi & Lin, 1967; Hayzer & Leisinger, 1982; Huang et al., 2016; Itoh et al., 2008; Kanatani et al., 1991; Katsuragi et al., 1986; Pacaud & Uriel, 1971; Powers & Snell, 1976; Simpson & Davidson, 1976; Zhang et al., 2009) and from our study (Table 1). For these literature studies, there are often only a few pH values tested. For example, the studies of GlpK (Hayashi & Lin, 1967) measured the stability at pH 5.0, 6.0, 7.0, and 8.0 and while it is apparent that the pH_50_ lies between 5.0 and 6.0, a more precise estimate is not possible. Thus, the data is consistent with our estimate of the GlpK pH_50_ 95% confidence interval of 5.65–5.70. Similarly, the pH₅₀ values obtained here are consistent with the data from half of these previous reports (Table 1). Specifically, the literature reports for AroG, GabD, GlpK, GshB, NanA, PanB, ProA, TesA, and TnaA are consistent with our proteomic-based assessments. Since four of the remaining proteins have a pH_50_ that is lower than the literature reports and five have a pH_50_ that is higher, there is no evidence of a systematic discrepancy between this proteomic-based study and the literature.

**Table 1:** The estimated pH50 values correspond to available literature data for *E. coli* proteins.

			Literature data​		
Protein​	Description​	95% CI of pH_50_ after 48 hr at 4 °C​	Literature pH_50_ range​	Incubation conditions​	Citation ​
AroG​	Phospho-2-dehydro-3-deoxyheptonate aldolase​	**[3.85, 5.18]**​	**5.0–6.0**​	8 min at 37 °C​	Simppson and Davidson
CodA ​	Cytosine/isoguanine deaminase​	[2.46, 4.05]​	5.0–6.0​	6 hr at 37 °C​	Katsuragi et al.​
GabD​	Succinate-semialdehyde dehydrogenase [NADP (+)] ​	**[4.15, 4.44]**​	**acid stable^1^**​	5 min at 37 °C​	Cozzani et al.​
GadB​	Glutamate decarboxylase B​	[5.02, 5.49]​	acid stable^2^​	24 hr at 4 °C​	Huang et al.​
GlpK​	Glycerol kinase​	**[5.65, 5.70]**​	**5.0–6.0**​	10 min at 25 °C​	Hayashi et al.​
GshB​	Glutathione synthetase​	**[3.83, 5.15]**​	**4.0–5.0**​	15 min at 42 °C​	Gushima et al.​
LdhA​	D-lactate dehydrogenase​	[4.60, 4.97]​	4.0–4.5​	1 hr at 30 °C​	Furukawa et al.​
NanA​	N-acetylneuraminate lyase​	**[4.63, 5.20]**​	**<5**​	15 min at 75 °C​	Aisaka et al.​
PanB​	3-methyl-2-oxobutanoate hydroxymethyltransferase​	**[1.0, 5.2]**​	**∼4.5**​	Not stated​	Powers et al.​
PheA​	Fused chorismate mutase/prephenate dehydratase​	[4.97, 5.64]​	3.9–4.5​	5 min at 37 °C​	Baldwin et al.​
PpsA​	Phosphoenolpyruvate synthetase​	[5.43, 5.50]​	∼5​	24 hr​	Berman et al.​
ProA​	Glutamate-5-semialdehyde dehydrogenase​	**[3.95, 5.01]**​	**4.5–5.0**​	8 min at 25 °C​	Hayzer et al.​
PssA​	O-phosphatidyltransferase​	[4.34, 5.04]​	5.5–6.0​	24 hr at 4 °C​	Zhang et al.​
PtrB​	Oligopeptidase B​	[5.41, 5.50]​	∼5.0​	15 min at 37 °C​	Kanatani et al.​
PyrF​	Orotidine-5'-phosphate decarboxylase​	[4.06, 5.70]​	∼6.0​	30 min at 25 °C​	Donovan et al.​
TesA​	Multifunctional acyl-CoA thioesterase I​	**stable**​	**acid stable^3^**​	30 min at 25 °C​	Pacaud et al.​
TnaA​	Tryptophanase​	**[4.89, 5.10]**​	**5.0–6.0**​	24 hr at 4 °C​	Burns et al.​
YihS​	Sulfoquinovose isomerase ​	[1.97, 3.94]​	5.0–5.5​	6 hr at 37 °C​	Itoh et al.​

We compared the estimated 95% confidence interval of the denaturation point to the stability assays reported in the literature. Literature values are reported as the two nearest experimental pH values to the presumed pH_50_. Bolding indicates consistency between the results reported here and the literature data.

Previous characterization of GabD had been performed at pH values as low as 5.0, with minimal observed decrease in stability (Cozzani et al., 1980). However, our approach was able to query a larger range of pH values and estimate a pH_50_ of 4.3. Previous characterization of purified TesA involved incubation at pH values as low as 2.0, with the observed maintenance of at least 60% stability relative to incubation at pH 7.0 (Pacaud & Uriel, 1971). This is consistent with our classification of TesA as stable due to its maintenance of normalized concentrations greater than 0.50 across all pH values.

Within the literature reports, the inconsistent treatment time of purified proteins is apparent. Specifically, incubation times range from 5 min to 24 hr and incubation temperatures from 4 to 75 °C. Surprisingly, the degree of similarity between the literature incubation condition and our own treatment of lysates at 4 °C for 24 hr does not appear to correspond to the degree of similarity between the observed pH_50_ values.

Thus, though there are only a few data points available for comparison, our proteomic approach appears to have reliably reproduced qualitative and quantitative assessments of pH stability gathered in traditional, laborious biochemical assays. The scarcity of data available for comparison highlights the need for this type of high-throughput analysis of protein pH sensitivity.

### Characterization of purified GltA is consistent with proteomic pH_50_

We selected citrate synthase (GltA) for further validation of the proteomic approach and for side-by-side comparison of stability and activity data. This essential protein catalyzes the synthesis of citrate in the tricarboxylic acid (TCA) cycle and is already known to be subject to allosteric inhibition by NADH and the downstream metabolite 2-oxoglutarate (Srere et al., 1963). To the best of our knowledge, the pH sensitivity of GltA has not previously been characterized, either on an activity basis or a structural basis.

**Figure 6:**
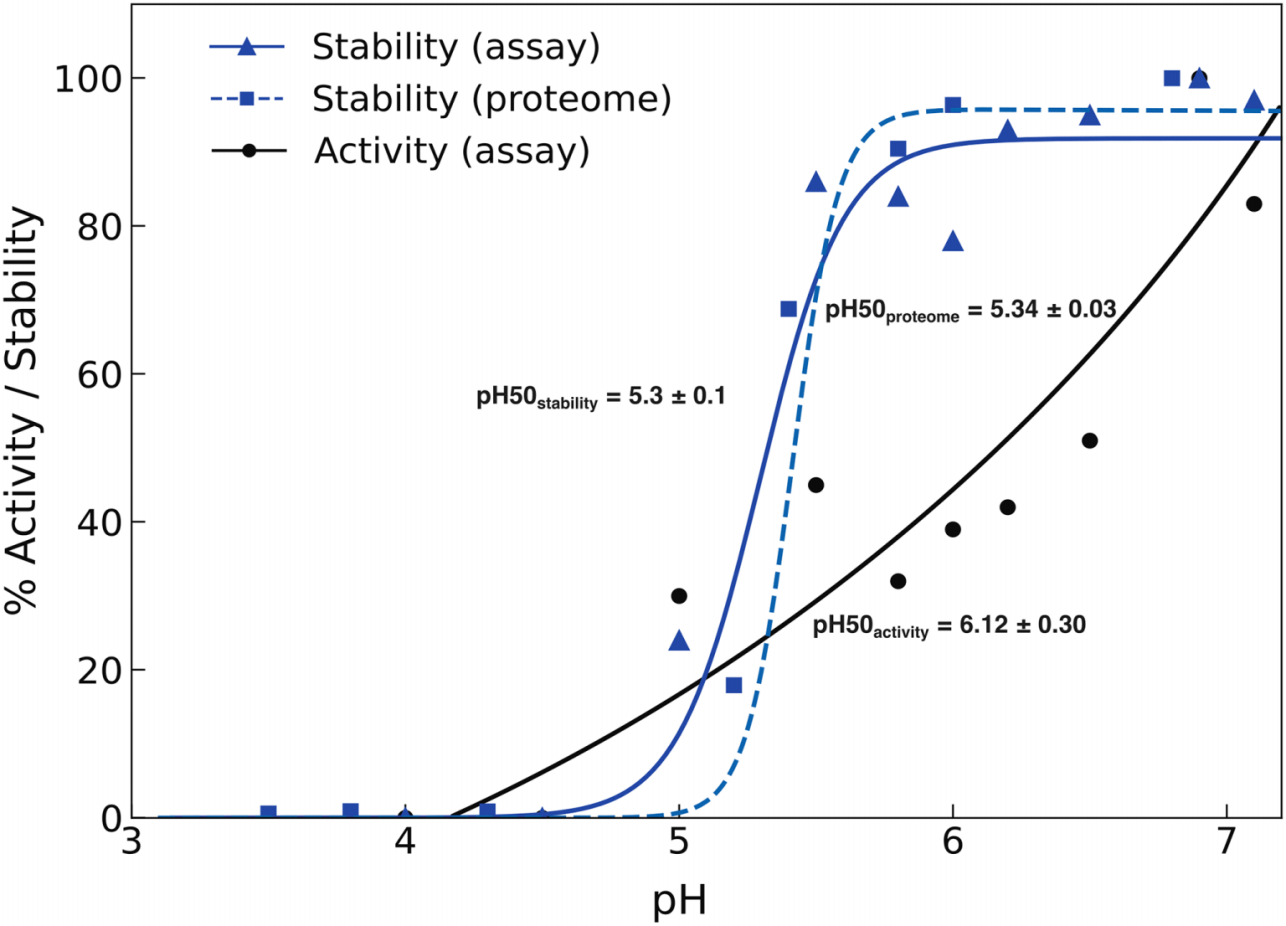
Proteomic-based assessment of GltA pH stability is consistent with stability assay measurements and is distinct from pH-dependent activity.​ Assay-based stability (blue triangles, solid blue fit) of purified GltA and proteome-derived stability estimates (blue squares, dashed fit) both exhibit a similar sigmoidal profile. Data are normalized to percent activity/stability relative to pH 7.0.​ Activity of purified GltA was measured in vitro (black circles, solid black fit).

GltA was cloned and purified as described above. For consistency with the proteomic data, the stability was assessed by incubating the purified enzyme at the indicated pH at 4 °C for 24 hr. Then the insoluble proteins were removed via precipitation. The amount of GltA protein remaining soluble was then quantified by measuring the activity in the supernatant at pH 6.9 according to a well-established assay (Srere et al., 1963) (Figure 6). The observed enzyme stability was relatively high at treatment pH values of 5.5 or higher, but at pH values of 4.5 or below, there was a complete loss of stability. Thus, the experimental assessment of the stability of purified GltA (pH_50_ 5.3 ± 0.1) is consistent with the proteomic-based estimate (5.34 ± 0.03), providing further support for our method.

This experimental assessment of GltA activity also provides the opportunity to directly contrast the pH dependence of enzyme stability to the pH dependence of enzyme activity. The impact of pH on enzyme activity can be assessed by performing the activity assay at various pH values at 25 °C (Figure 6). Contrastingly, as demonstrated above, the assessment of stability uses a single assay pH to query the retention of enzyme stability following treatment. The difference between GltA stability and activity dependence on pH is apparent. Specifically, while the enzyme mainly retains its soluble form at pH values as low as 5.5, its activity is halved (relative to pH 7.0) at a pH value of 6.5. This demonstrates the importance of explicitly defining which protein characteristics are being measured and described. GltA’s acid sensitivity may limit *E. coli*’s ability to maintain efficient TCA cycling under acidic fermentation conditions, potentially reducing the ability to replenish essential metabolic intermediates or productivity in processes such as succinic acid production. This is also consistent with the observed shift in metabolic flux toward lactic acid during fermentation at low pH (C. Wu et al., 2026). The greater pH sensitivity of GltA activity relative to stability is consistent with general protein behavior. Specifically, this is likely a result of the dependence of activity on the protonation states of a small number of active-site residues and local electrostatic networks, whereas overall protein stability is buffered by distributed interactions across the entire protein structure (Garcia-Moreno, 2009; Talley & Alexov, 2010).

### pH sensitivity differs by subcellular location

The data obtained here provides insight into general trends of protein pH sensitivity, such as variation according to sub-cellular location. Mateus et al previously performed a similar analysis on *E. coli* protein thermostability and location, with the conclusion that periplasmic proteins had increased temperature tolerance (Mateus et al., 2018). We flagged proteins according to their known subcellular compartment, as specified by UniProt, and binned according to their estimated pH_50_ values (Table 2). Significant differences in distribution according to location were observed.

**Table 2:** Proteins pH stability significantly varies according to subcellular location.

pH_50_	Cytoplasm (*n* = 354)	Inner membrane (*n* = 178)	Periplasm (*n* = 79)	Outer membrane (*n* = 21)
Stable	23^a^ (6%)	12^a^ (7%)	20^b^**(25%)**	2^a,b^ (9.5%)
< 4.0	17^a^ (5%)	7^a^ (4%)	10^a^ (13%)	-
4.0–4.5	36^a,b^ (10%)	7^a^ (4%)	12^b^ (15%)	1^a,b^ (4.8%)
4.5–5.0	77^a^ (22**%**)	14^b^ (8%)	13^a,b^ (16.5%)	2^a,b^ (9.5%)
5.0–5.5	141^a^**(40%)**	49^b^ (28%)	13^b^ (16.5%)	8^a,b^ (38.1%)
5.5–6.0	58^a^ (16%)	81^b^**(45%)**	11^a^ (14%)	6^a,b^ (29.6%)
6.0–6.8	2 (1%)	8 (4%)	–	2 (9.5%)
Median pH_50_	5.14	5.52	4.88	5.46

The values shown are the counts and percentages relative to the total number of proteins in each subcellular compartment. We considered pH_50_ values with std <=1. Superscript letters indicate statistically significant differences between subcellular locations within the same pH bin (pairwise chi-square tests with Benjamini–Hochberg false discovery rate correction, *p* < 0.05) with statistically significant percentages highlighted in bold. *P*-values are provided in supplementary Table S4.

Cytoplasmic proteins were enriched in the moderately acidic range (pH_50_ = 4.5–5.0, 5.0–5.5) relative to those from the inner membrane and periplasm. Specifically, 62% of cytoplasmic proteins had a pH_50_ in the range of 4.5–5.5, compared to 36% and 33%, respectively. Inner membrane proteins were enriched at higher pH_50_ values, particularly in the pH_50_ 5.5–6.0 bin (45%), relative to less than 20% for the cytoplasmic and periplasmic proteins. No significant differences were observed between peripheral membrane proteins and transmembrane proteins (Table S5). Finally, the periplasmic proteins had higher representation in the bins representing acid tolerance: especially in the “stable” category. Note that no significant differences were observed for outer membrane proteins, presumably due to the relatively small sample size (*n* = 45).

The apparent acid stability of the periplasmic proteins is consistent with Liu et al.’s study where periplasmic proteins were shown to be stable under acid and other harsh conditions (Liu et al., 2004). Specifically, proteins in periplasmic extracts retained solubility, evidenced by visualization on 2D-gel after treatment for 15 min with 0.5 M HCl, while non-periplasmic extracts were highly aggregated.

Membrane proteins were comparatively underrepresented in our dataset, which should be considered when interpreting localization-dependent trends in pH stability. Among proteins with a standard deviation of pH_50_ ≤ 1, we recovered only 18% of UniProt-annotated inner membrane proteins and 21% of UniProt-annotated outer membrane proteins, compared to 51% of cytoplasmic and and 44% periplasmic proteins in the database. This reduced sampling of membrane-associated proteins is consistent with expectations, as no detergent was included in the lysis conditions, limiting the efficient solubilization and recovery of hydrophobic membrane proteins.

### Acid enrichment of specific proteins

As described above, we were able to estimate a pH_50_ value for more than 90% of the proteins detected in our experimental condition. Approximately 9% of these proteins were deemed “stable”, such as TesA. Some proteins showed fluctuations in the normalized concentration that prevented either their classification as stable or the assignment of a pH_50_ value. Seventeen of these proteins had sparse or noisy data and were classified as “other”. Surprisingly, there were two proteins that showed increased abundance/concentration as the treatment increased in acidity.

**Figure 7:**
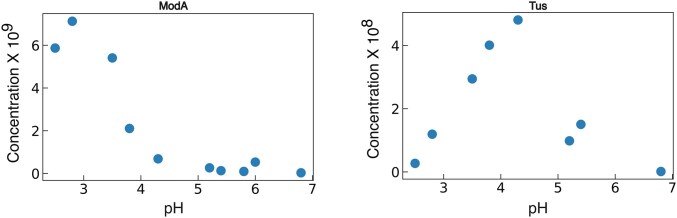
Abundance profiles of ModA, a periplasmic ABC transporter that mediates high-affinity uptake of molybdate, and Tus, a DNA replication terminus site-binding protein, both showing increased abundance under low pH conditions.

Specifically, ModA, the periplasmic binding protein of the molybdate ABC transporter, became increasingly abundant with decreasing pH (Figure 7). Tus also exhibited a distinct stability profile, with abundance peaking near pH 4.0 (Figure 7). These results suggest that some proteins may not only possess enhanced stability under acidic conditions but also are enriched relative to the baseline treatment. One possible explanation for this enrichment is increased accessibility of the soluble protein fraction to the proteolytic treatment preceding LC/MS.

### Correlation of pH_50_ with isoelectric point, melting temperature, and molecular weight

The large amount of data regarding protein temperature sensitivity enables comprehensive analyses, such as those presented by Pinney et al., (2021). However, there is relatively less data regarding protein pH stability. Here, we performed a cursory comparison of the estimate pH_50_ values with other available protein properties. Specifically, we examined possible correlations between pH_50_ values and isoelectric point (pI) and molecular weight (MW), both obtained from UniProt (The UniProt Consortium et al., 2025) and mainly predictable based on protein sequence. Linear regression analysis revealed no meaningful associations (Figure S2).

We also compared our set of estimated pH_50_ values to the estimated *T_m_* for each protein. Three studies have recently queried the temperature stability of the *E. coli* proteome^12–14^; our analysis considered each of these independently (Figure S3). However, again no meaningful trends were observed.

## Discussion

In this study, we developed and validated a framework for proteome-wide assessment of acid stability with *E. coli* as the model organism. Our work addresses a critical gap in the understanding of protein stability under acid stress. Studying pH-dependent stability is particularly challenging because intracellular pH is tightly regulated and decoupled from external pH. Designing experiments that account for this distinction is therefore essential for accurately estimating protein pH-denaturation profiles in the cell.

More than half of the proteins detected in our study overlapped with those identified in previous proteome analyses conducted under similar conditions (Jarzab et al., 2020; Schmidt et al., 2016) showing good coverage of the *E. coli* proteome. Based on the normalized concentrations we estimated the pH_50_ and its confidence interval. Notably, confidence intervals were not reported for the melting points in previous studies of protein thermostability (Jarzab et al., 2020; Leuenberger et al., 2017).

Validation against existing literature and experimental data supports the reliability of our method in estimating protein pH stability. For the subset of 18 proteins with prior stability enzyme characterizations, our pH_50_ estimates aligned with half of the reports. The discrepancies with the other reports may stem from heterogeneous experimental conditions in prior studies, such as variable incubation times (5 min–24 hr) and temperatures (4–75 °C), compared to our study with incubation for 48 hr at 4°C. Further validation with purified citrate synthase (GltA) confirmed consistency: the proteomic pH_50_ (5.34 ± 0.03) matched experimental assessment (5.3 ± 0.1) (Figure 6). The scarcity of comparable data pH stability data in the literature on *E. coli* proteins highlights the value of our high-throughput approach to studying protein stability.

In studying the association of stability with respect to subcellular location, we found that periplasmic proteins were significantly more stable compared to other locations. This is consistent with protein stability studies (Jarzab et al., 2020; Mateus et al., 2018) and studies on acid and other stressors (Liu et al., 2004).

Two proteins, ModA (molybdate ABC transporter periplasmic binding protein) and Tus (DNA replication terminator), apparently increased in abundance with decreasing pH, peaking at low values. This enrichment may arise from enhanced proteolytic accessibility in the soluble fraction or pH-induced conformational changes promoting solubility, rather than true stabilization. Such behaviors expand beyond simple destabilization models and warrant mechanistic follow-up, potentially linking to acid stress responses such as organic acid tolerance (Royce et al., 2014).

An important future direction leveraging this dataset to develop predictive models of protein pH stability. Such models could streamline enzyme engineering by identifying unstable proteins that could be modified to improve the robustness or even predict protein sequences and structures that are associated with highly stable proteins. Incorporating this dataset as an additional training dimension could enhance existing machine learning models of protein pH activity (Gado et al., 2023), improving predictive accuracy across diverse proteins.

Finally, this approach is broadly applicable. It can be extended to other stressors such as solvents or salinity, and to diverse organisms, providing a versatile framework for mapping proteome stability under suboptimal conditions.

## Supplementary Material

kuag016_Supplemental_Files

## Data Availability

The datasets and computer code in this study are available in the following databases: Protein MS data: PRIDE PXD0074625. Computer code: GitHub https://github.com/cypetee/Proteomics-pH-stability.
