# Supplementary material for: Proteome-wide analysis of protein stability in Escherichia coli under acid stress
Source: J Ind Microbiol Biotechnol. 2026 Jul 1;53:kuag016. doi: 10.1093/jimb/kuag016 (PMC13339084; doi:10.1093/jimb/kuag016)
Supplement: kuag016_Supplemental_Files [file kuag016_supplemental_files.zip › Supplementary Figures for comment.docx]

Supplementary Figures

 
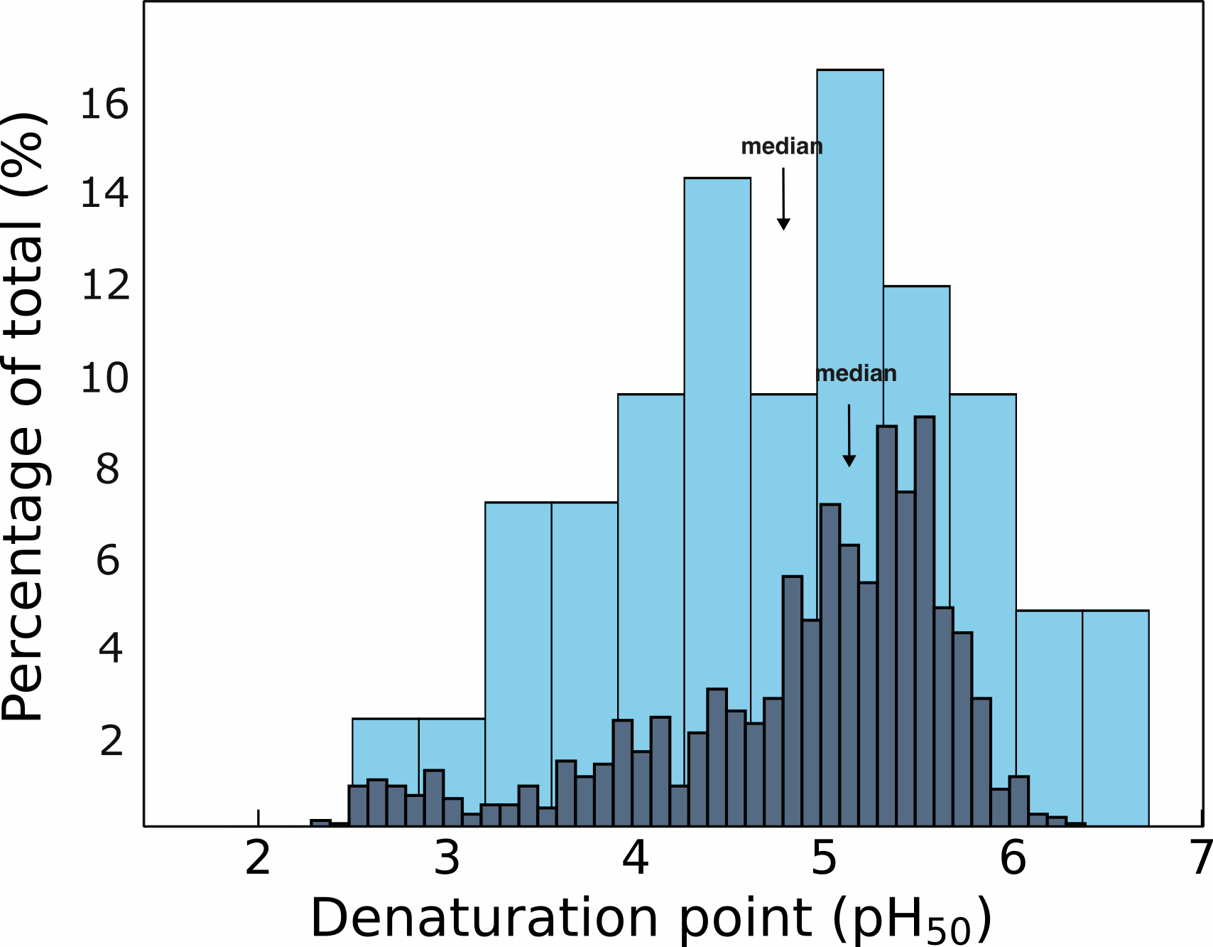


**Fig. S1.  Distribution of protein denaturation points (pH₅₀) in enteric bacteria.**​

Frequency distributions of pH₅₀ values derived from literature studies for enteric bacterial proteins (*n* = 42; light blue) and from this proteomics study (*n* = 1,458; dark gray). Arrows indicate the median pH₅₀ for each dataset (literature: 4.65; proteomics: 5.11)

**Alt text:**

An overlaid histogram comparing the denaturation point (pH_50_) distributions of two protein datasets. The x-axis shows denaturation point (pH_50_, range approximately 2–7) and the y-axis shows percentage of total proteins (0–17%). Light blue bars (wider bins) represent literature data for enteric bacterial proteins with a distribution centered around pH 4.5–5.0 and a median annotated by an arrow near pH 4.7. Dark gray bars (narrower bins) represent a dataset from this study with a right-shifted distribution peaking around pH 5.2–5.5.


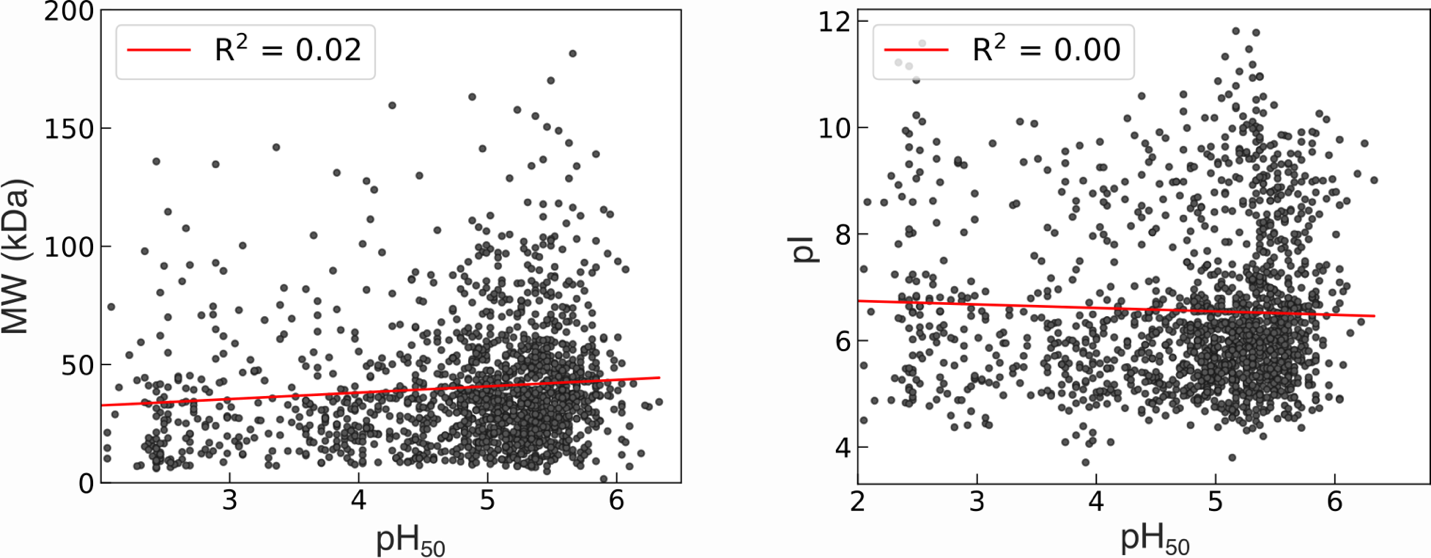


**Fig. S2**. **Protein isoelectric point (n=1675) and molecular weight (n=1675) showed no significant correlation with pH_50_**. The red line represents the regression line. For both correlations, the Pearson’s correlation coefficient was < 0.02. Theoretical isoelectric points and molecular weights were calculated based on amino acid sequences of proteins identified from the UniProt database.​

**Alt text:**

Two side-by-side scatter plots evaluating potential correlations between protein pH_50_ denaturation point and two physicochemical properties. Left panel: molecular weight (MW, kDa; y-axis, range 0–200) plotted against pH_50_ (x-axis, range approximately 2–6.5). Gray filled circles represent individual proteins (n ≈ 1,511). A red linear regression line is nearly flat with slight positive slope; R² = 0.02, indicating no meaningful correlation between pH_50_ and protein molecular weight. Right panel: isoelectric point (pI; y-axis, range approximately 3.5–11) plotted against pH_50_ (x-axis, range approximately 2–6.5). Gray filled circles cluster densely between pI 4–8 and pH_50_ 4.5–5.5. A red regression line is nearly horizontal; R² = 0.00, indicating no correlation between pH_50_ and pI.


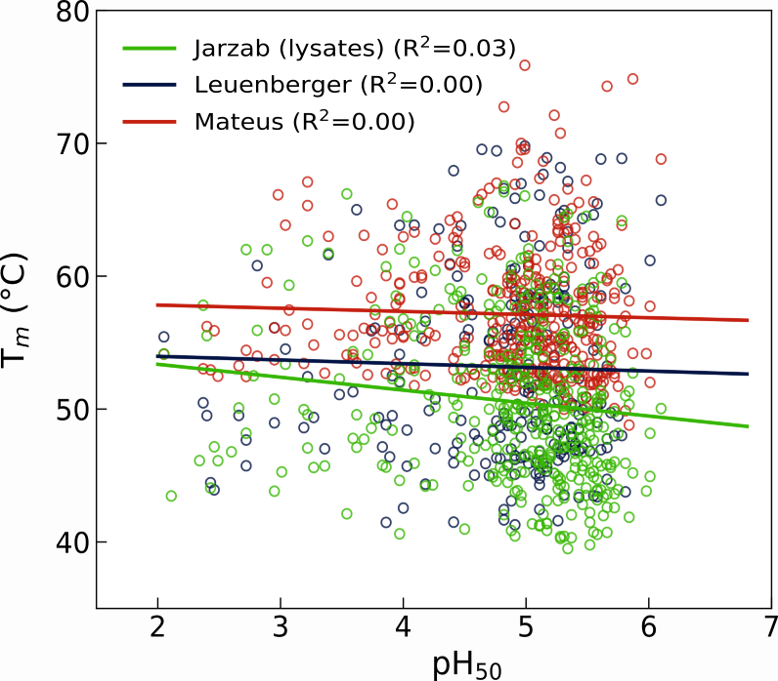


**Fig. S3.** **There was no correlation between the estimated pH50 and previously-reported melting point (Tm).** Melting temperature data on E. coli enzymes was collected from 3 independent thermostability studies (Leuenberger, n = 249; Mateus, n = 404, Jarzab, n = 409).The Pearson’s correlation coefficient was < 0.03 in all cases.

**Alt text:**

A scatter plot comparing acid denaturation point (pH_50_, x-axis, range 2–7) melting temperature (Tm, degrees Celsius; y-axis, range approximately 40–80°C) for proteins common to this study and three published thermal proteome profiling datasets. Open circles are colored and overlaid for three reference studies: green circles for Jarzab et al. (lysates), dark navy circles for Leuenberger et al., and red circles for Mateus et al. Three linear regression lines are drawn: a green line for Jarzab with slight negative slope (R² = 0.03), a dark navy line for Leuenberger (R² = 0.00), and a red line for Mateus (R² = 0.00).
